# May Artificial Intelligence take health and sustainability on a honeymoon? Towards green technologies for multidimensional health and environmental justice

**DOI:** 10.1080/11287462.2024.2322208

**Published:** 2024-03-11

**Authors:** Cristian Moyano-Fernández, Jon Rueda, Janet Delgado, Txetxu Ausín

**Affiliations:** a Institute of Philosophy, Spanish National Research Council, Madrid, Spain; b FiloLab Scientific Unit of Excellence, University of Granada, Granada, Spain; c Department of Philosophy 1, Faculty of Philosophy, University of Granada, Granada, Spain; d Institute of Philosophy, Spanish National Research Council, Madrid, Spain

**Keywords:** Artificial intelligence, sustainability, health and environmental justice, long-term, nonanthropocentrism

## Abstract

The application of Artificial Intelligence (AI) in healthcare and epidemiology undoubtedly has many benefits for the population. However, due to its environmental impact, the use of AI can produce social inequalities and long-term environmental damages that may not be thoroughly contemplated. In this paper, we propose to consider the impacts of AI applications in medical care from the One Health paradigm and long-term global health. From health and environmental justice, rather than settling for a short and fleeting green honeymoon between health and sustainability caused by AI, it should aim for a lasting marriage. To this end, we conclude by proposing that, in the upcoming years, it could be valuable and necessary to promote more interconnected health, call for environmental cost transparency, and increase green responsibility.

**Highlights**

- Using AI in medicine and epidemiology has some benefits in the short term.
- AI usage may cause social inequalities and environmental damage in the long term.
- Health justice should be rethought from the One Health perspective.
- Going beyond anthropocentric and myopic cost–benefit analysis would expand health justice to include an environmental dimension.
- Greening AI would help to reconcile public and global health measures.

## Introduction

Artificial Intelligence (AI) tools used for healthcare and public health have considerably expanded in the last few years (Murphy et al., 2021). AI systems developed for the COVID-19 disease early detection and prognosis might be seen as a breakthrough for health from the perspective of justice, because they have increased efficiency and streamlined care by prioritising the allocation of healthcare resources (Adamidi et al., 2021). Similarly, contact tracing tools have been used to promote public health (Jacob & Lawarée, 2021). However, some ethical biases have been observed in these AI applications (Delgado et al., 2022; Röösli et al., 2021). Hence, how should these AI applications be discussed from a commitment to justice?

Dealing with such a question involves not only considering the social determinants of health, but also the ecological reversals of AI used in health (Richie, 2022). Despite the few existing analyses on the carbon footprint and the energy consumption of AI processes (Hagendorff, 2022), several researchers have already warned of the potential environmental damage that may be caused if its development continues to increase (Dhar, 2020; Heilinger et al., 2023; Mulligan & Elaluf-Calderwood, 2022; van Wynsberghe, 2021; Wu et al., 2022).

Reviewing the most prominent studies on this topic, we aim to rethink the global health trade-offs that may derive from AI applications to healthcare and public health. We wonder about the moral balance of contributions of AI applied to health care and epidemiology from a concern for health and environmental justice. AI applications can be shaped by social, economic, political, and cultural factors, and they also lead to environmental impacts. Thus, it is necessary to unravel the contrasts that emerge from different approaches to health and conceptions of justice.

The aim of this paper is thus threefold. First, we attempt to offer a reasonable account of health justice from the One Health paradigm. We consider here that the sustainability of AI-based applications should be addressed in the long-term and from systemic analysis, in order to properly assess the health benefits provided by them. Second, despite the difficulties in determining the environmental cost of AI systems, some tools and methods developed in the last few years can be useful to account for these impacts, so we list some of these. We point out that when the global cost–benefit calculation in health is discussed from myopic and anthropocentric lenses, a more relational framework of justice to embrace ecological concerns should be included. Third and finally, beyond understanding AI for health and AI sustainability as an unsurmountable dichotomy, efforts should be made to reconcile them when exploring the ethical impacts of AI. To this end, we will conclude with a brief list of ethical recommendations that may serve as tipping points for advocating green and just AI applied to the field of health.

## One health

The main question we aim to address is: to what extent is it fair that AI development, and in particular its health applications, may paradoxically shorten health? To unravel this issue, the first step is to explore why health or its absence is a matter of justice.

We may start by broadly defining what we understand by “health justice”. Health justice has been defined by some authors as the constitution of universal ethical health norms and shared global and domestic responsibility for health (Ausín, 2021). Research for health justice aims to reduce health disparities between and within countries and ground ethical obligations between different actors (Prah Ruger & Horton, 2021). Health justice consists of recognising the suffering of many individuals as health injustice due to systemic and socio-structural causes, instead of as an unfortunate, natural phenomenon or as a result of personal choices (Pratt, 2021). For example, instead of viewing the recent COVID-19 pandemic as a tragedy or a natural disaster, it is challenging but necessary to understand the pandemic as a reflection of social injustice on a grand, global scale. Recognising the social injustices surrounding this pandemic or future diseases and vulnerabilities that may emerge is also an important opportunity to understand the long-standing links between health and environmental justice.

Environmental justice has been mainly conceived as a framework to fairly allocate environmental resources and impacts among unequal people (Whienhues, 2020). While some authors have used the concepts of environmental justice and ecological justice as synonyms (Low & Gleeson, 1998), others have made the effort to see their different nuances (Schlosberg, 2007). According to the latter, in environmental justice only human beings are part of the community of justice while nonhuman beings and nature are somehow reified. In contrast, in ecological justice, nonhuman beings and nature are also included within the community of justice since they do not matter only because of what benefits or harms human beings, but they matter in themselves.

In this article we stay away from these discussions between environmental and ecological justice because our aim here is not to disentangle whether nonhuman beings and nature have intrinsic value and should be a subject of justice, or not. Rather, our purpose is to understand how human health depends on environments with biodiversity and ecosystem functionalities that are not severely disrupted by us, and for this it is not necessary to grant intrinsic value to nonhuman beings and nature (Moyano-Fernández, 2022).

Thus, we are aligned with One Health perspective, which seeks to learn from the intersection between social, environmental, and animal interfaces, considering these as interdependent matrixes with connected health (Acharya et al., 2021). Human societies are built on the use and consumption of nonhuman animals, and nonhuman animals can significantly influence ecosystem dynamics, which in turn will affect human health (Halabowski & Rzymski, 2021; Sebo, 2022). For example, the most reasonable explanation for the COVID-19 outbreak was a zoonotic cause, which means that this disease was due to contact between humans and nonhuman animals (Andersen et al., 2020). Wildlife trade, mink farming, meat production, and hunting activities, among others, are major drivers of potential zoonotic transmission because they can increase the reservoirs for viruses (Halabowski & Rzymski, 2021), so the susceptibility of not only humans but also wild, domestic and livestock animals must be considered when health is in the spotlight of justice.

Avoiding overexploitation of nonhuman beings makes it easier for ecosystems to maintain good biodiversity and thus ecological functionalities beneficial to our health in the long term (Moyano-Fernández, 2022; Sebo, 2022). Pollution, global warming, droughts, or increasing zoonotic diseases are detrimental health reversals caused by environmental degradation and unsustainable use of nature (Rabinowitz & Conti, 2013). Some of these effects are not immediately noticeable, but only in the long term.

Hence, we need to protect healthy ecological processes in order to ensure human development and flourishing (Shockley, 2022). Committing to health justice implies looking systemically at the long-term effects of our relationship with nature. From now on, when we refer here to health justice we are assuming that a global and One Health perspective is necessary, and thereby health justice becomes intertwined with environmental justice. Therefore, in this work, we prefer to use the term health and environmental justice to emphasise the interconnectedness of the two.

Now, once agreed that health concerns must encompass ecological care, the next question to be addressed is how AI systems, including those used for health, affect the environment. This question is crucial for our bioethical study because if the development of AI used for health generates long-term damage to global health due to its energy demand and its respective environmental cost, then we are faced with an ethical problem that is difficult to solve without accepting moral tradeoffs.

## Short-term versus long-term benefits

Currently, it is well-reported that AI applications in health systems have the potential to provide beneficial outcomes at both individual and public levels by early detection and diagnosis, prognosis, and tracing the spread of diseases (Briganti & Le Moine, 2020; Glauner et al., 2021; Matheny et al., 2020). Actually, the concept of “One Digital Health”, understood as the operationalisation of One Health through digitalisation, has already been proposed (Ho, 2022). Even some studies have noticed that AI tools and systems used in healthcare, specifically in telemedicine, have the potential to further reduce greenhouse gas emissions by up to 80% (Purohit et al., 2021; Wolf et al., 2022). This would exemplify how AI is used to help achieve sustainability purposes.

However, AI may not always be in line with sustainability, and this needs to be taken into account (Moyano-Fernández & Rueda, 2024; van Wynsberghe, 2021). The prospective benefits for individual and public health sometimes have environmental reversals that threaten global health through resource depletion, accelerating climate change, and biodiversity loss (Dhar, 2020; Wu et al., 2022). AI technologies can have a significant ecological impact on the biosphere cycles: they are highly demanding in terms of energy, materials, and other resources. These impacts are not particularly visible in the health sector, since the laudable aim of saving lives now (or widening access to save lives now) may eclipse the potential risks to future generations through environmental damage (Samuel et al., 2022).

Despite the difficulties in determining the environmental impact of AI systems, some tools and methods developed in the last few years can be useful to account for these impacts. Ligozat et al. (2022) enumerate the available tools and methods for assessing AI’s environmental impacts. Some of them are focused on reporting the direct impacts of an AI service, while others are rather focused on its indirect impacts. In the following table, we summarise and update some of them (Table 1).

**Table 1.** Summary of relevant available methods for measuring AI’s environmental impact.

Target	Scope of AI’s assessment	Tools/Methodologies
Reporting measured AI’s energy consumption and the associated carbon footprint	*AI service*: restricted to the equipment used by the AI (direct impacts included)	Integrated tools, e.g. *Experiment Impact Tracker* (https://github.com/Breakend/experiment-impact-tracker) (Henderson et al., 2020), *Carbon Tracker* (https://github.com/lfwa/carbontracker) (Anthony et al., 2020), or *CodeCarbon* (https://codecarbon.io/), or online tools, e.g. *Green Algorithms* (http://www.green-algorithms.org/) (Lannelongue et al., 2021), or *ML CO2 impact* (https://mlco2.github.io/impact/#compute)
Assessing the society’s costs and the economic benefit ratio of an AI used for the Green method (i.e. AI used to reduce energy consumption or to benefit other environmental indicators)	*AI solution:* a complete application using AI (indirect impacts included)	Reporting a wide range of aspects (societal and behavioural changes, and economic gains) of Foundation Models based on acceptance of their homogenisation and emergence (Bommasani et al., 2021). Recently developed a Holistic Evaluation of Language Models methodology (Bommasani et al., 2023)
Assessing the more-than-economic impacts of AI from a life cycle assessment analysis	*AI solution and services*: going beyond the use phase and including the production, the end of life, etc. (direct and indirect impacts)	Methodologies based on the Life Cycle Assessment framework (Ligozat et al., 2022)

These strategies might help to illustrate the tradeoffs of using AI in healthcare. By measuring the AI’s multi-level impacts, it is possible to better assess the cost/benefits relationship of AI used to improve health in the long term and from a systemic view.

As noted in the table, when AI’s environmental costs are studied, more-than-carbon tunnel vision must be embraced (Deivanayagam & Osborne, 2023). Although some authors have concluded that a few AI applications involving telemedicine can reduce the carbon cost of healthcare (Tsagkaris et al., 2021), some others remain sceptical about using the carbon footprint as a primary metric (Richie, 2022). On the one hand, although there is a bilateral positive causal bond between healthcare expenditures with carbon dioxide emissions (Ahmad et al., 2021), healthcare carbon expenditure does not necessarily translate into better medical quality of life because unaddressed social determinants of health and biases could affect the outcomes (Bilgili et al., 2021; Delgado et al., 2022). Additionally, beyond carbon dioxide emissions of AI applied to health, this technology also has adverse indirect impacts on the material environment (where data centres are constructed), the unsustainable extraction of minerals for technological components, and e-waste (discarded electronic appliances) disposal (Samuel & Lucassen, 2023).

Furthermore, a green AI transition towards more accurate models –by using smaller and more carefully curated data sets (WHO, 2021)– or reliance on renewable energies would not guarantee a zero cost to the environment for two main reasons. First, the Jevons paradox should be considered. The Jevons paradox states that, in the long run, an increase in resource use efficiency will generate a rise in resource consumption rather than a decrease (Brockway et al., 2021; Ellegaard Fich et al., 2022). Digital pollution is expected to grow despite improvements in the efficiency of digital systems, since these improvements and the consequent lowering of costs will increase demand and consumption (Blair, 2020). This rebound effect may also be called AI’s “third-order impacts” (Ligozat et al., 2022). Second, extraction of materials, transport, and green infrastructures –like solar panels, artificial dams, or wind turbines– can cause toxic emissions, habitat deterioration, physical damages, and changing behaviours of nonhuman species, leading to a loss of biodiversity (Gasparatos et al., 2021).

## Green honeymoon

Acknowledging the complex green cost of AI may help to rethink the ecological and multi-level impacts of AI used in medicine and health from a long-term^1^ and systemic perspective, where the environmental effects might also damage the health of some human communities, mainly those belonging to a middle and low-income group or the most marginalised by inequalities of power (Holzmeyer, 2021), future generations and more than human species.

As we have seen so far, health and environmental justice implies adopting a concern for long-term health. This entails caring for nature and its interdependencies, even as we strive to reduce health injustices, since this struggle sometimes results in environmental impacts. Health and environmental justice mediated by the One Health view aims to be critical of strong anthropocentrism, which does not even recognise our eco-dependence on nature, and shortsighted cost–benefit calculus in environmental assessments (Muradian & Gómez-Baggethun, 2021).

Modern industrialised healthcare as practiced in most high-income countries (and considered aspirational elsewhere) was derived from the same worldview that has mortgaged the health of future generations (Wardrope, 2020). Similarly, sustainable global health, focused on delivering health services with a low environmental burden to underserved populations in resource-poor areas of the world, has also been considered a moral duty by some scholars (Pratt, 2022).

These claims for sustainable health promotion and care are connected to our proposal, as they insofar appeal to the relationship between human and environmental health. However, the philosophical goal of our contribution here is more concise. We highlight the incoherence of some AI methods used in medicine to improve health when they cause environmental damage that will negatively affect global health in the long run. We consider this inconsistency important because it would seem to emerge from two deep-rooted narratives in tension. While AI-based health applications are dedicated to improving medical practice and providing short-term benefits to human lives as a public health issue, the challenge of maintaining global, sustainable, and interdependent health requires greater attention.

A commitment to health from a justice perspective can only be credible if it addresses both of these approaches, while striving to reduce conceptual dichotomies and ethical tensions. It might seem that if reliance on AI systems by medicine and public health implies environmental impacts that cannot even be offset by a green transition, then we are faced with a dilemma of choosing between: (a) prioritising the lives of communities of present generations whose health may benefit from AI's health advances, or (b) prioritising the lives of present and future communities whose health may be damaged by AI's environmental impact. In short, it seems that a health and environmental justice approach to address AI’s sustainability used in health pushes the choice of ensuring health horizontally or vertically on the timeline.

We consider that one of the main trade-offs of the AI used in health is its environmental cost, which can unjustly damage health in the long term, because its design, development, and application do not arise from unanimous voices (but they arise from the most industrialised and socio-economically powerful groups) and its effects do not impact equally on the entire present and future population (but they are more concentrated on some groups, mostly on future generations).

Thus, AI used for health becomes a paradox. It would seem that the idea that AI-powered applications developed for health can only embark, at best, on a fleeting honeymoon with sustainability. A green honeymoon whose days are numbered and with divorce on the horizon.

## Alternatives to mitigate the health trade-offs of AI used for health

Nevertheless, efforts can be made to reconcile health and sustainability in a durable marriage through green digital technologies. The paradox of AI used in health previously pointed out can be addressed by suggesting some conceptual alternatives framed by health and environmental justice. Here, we summarise a list of three suggestions that may serve to this purpose:

*Interconnected health*. Recognising the interdependencies of factors affecting health, like socioeconomic and ecological conditions. This implies taking a long-term and systemic perspective based on valuing the community itself (not always reducible to its individuals) when analysing health trade-offs (Wardrope, 2020). To put this alternative into practice, more interdisciplinary scientific research aligned with the One Health paradigm would be needed. A deconstruction of the dominant neoliberal narratives on health could offer a critique of the “innovation” sold through the new technologies production and inspire new approaches from which health is conceived more as an interconnected work-in-progress than as an atomised outcome. Committing to the alternative of interconnected health would imply, for example, policy measures of reviewing the different types of health interventions based on AI, focusing on the tension between growing investments in ever more individualised treatments in downstream clinical settings, on the one hand, and underinvestment in upstream social determinants of health and the public health sector, on the other (Holzmeyer, 2021).
*Environmental cost transparency.* Making costs and damages visible is necessary to settle the justice accounts. For example, some have suggested making visible the carbon footprint of AI-powered devices in the form of a “Tech Carbon Footprint Label” (Brevini, 2020). With such measures, regulators and the public could be properly informed about the implications of adopting each smart technology. However, as we pointed out in Section 3, the environmental costs of AI go beyond its carbon dioxide emissions. Environmental cost transparency should also involve making the regeneration cycles of natural resources visible, because these are sometimes slow and require time before they are used or exploited. Health and environmental justice should not only be about allocating resources fairly, but also about identifying what we should and should not yet allocate. In this regard, for example, Richie has proposed a normative principle, *resources conservation* (Richie, 2022), to guide environmentally sustainable healthcare.
*Green responsibility*. Multidimensional health and environmental justice implies consolidating a new responsibility for the environmental impacts of AI used in health. Traditionally, healthcare ethics has relied on individualised notions of risk (Samuel & Richie, 2022), but it must be broadened to consider collective responsibility for environmental sustainability. This implies collectively assuming, first, a fair distribution of costs and benefits, and second, a precautionary principle (Martuzzi & Tickner, 2004). The call for social responsibility for global health and environmental sustainability has long been advocated not only to compensate those most affected, but also to prevent harm. This is embracing a commitment to ex-ante and ex-post responsibility (Young, 2011). A green responsibility could minimise the environmental and global health trade-offs of AI used in health causes, for example, through ex-post measures such as (economic or health services coverage) compensations to those communities most affected by AI's environmental externalities, or through preventive measures such as deploying participatory policies whereby different voices are listened to, especially those less represented or with less power to influence (Kwete et al., 2022; Santos Barquero et al., 2021). The governance of health and environmental justice should not be unanchored from a political reconciliation in which different actors are taken into account when analysing the pros and cons of AI tools used in health.

## Conclusion

The population’s health is intimately linked to the health of the environment and other animals, as stated in the One Health paradigm. The use of AI by healthcare and epidemiology may have short-term benefits for humans, if social determinants of health and other biases in data capture, processing, and replication are addressed. This goal can be defended from a health and environmental justice view of AI that is oblivious to moral concerns about nature. However, human health is interconnected with healthy and biodiverse ecosystems. Hence, we have suggested that this perspective should be in dialogue with a broader approach to global health concerned with systemic and long-term effects, and critical of anthropocentrism. Even if the carbon footprint is integrated into AI environmental analyses, the Jevons paradox, biodiversity loss, or nonhuman animals damage are threads that can slip away from a short-term human-centred focus. For this reason, it would be desirable that health and environmental justice expands the scope to include new narratives of health, more transparency of the multidimensional AI impacts on ecosystems, and a green and collective responsibility. Thus, the balance for justice that the mass production and use of AI in health causes can be more properly addressed.

## Note

1 By long-term environmental impacts, we mean those that become an existential threat after a period of time longer than five years. According to one of the latest reports published by the World Economic Forum (2021), by long-run risks we may think of biodiversity loss, natural resource crisis, or climate action failure, among other events. Some scholars have led their research works on biological conservation or global health using explicitly long-term methods, understood as scenario analyses over a time horizon of five or more years (Barroso et al., 2020; Chen et al., 2020; Rai et al., 2021). Thus, when in this article we call for adopting a long-term perspective, we are referring to assessing the AI effects over a similar timeframe, because this is when new environmental or health impacts might become visible or even measurable, according to the above-mentioned studies.
